# 222. Effectiveness of the Original BNT162b2 Messenger RNA Vaccine Against COVID-19 Omicron Infection in Children Aged 6 Months to 4 Years in Japan

**DOI:** 10.1093/ofid/ofae631.080

**Published:** 2025-01-29

**Authors:** Yuta Aizawa, Yugo Shobugawa, Jun Tachikawa, Tatsuki Ikuse, Chansu Shin, Takao Ikarashi, Takayoshi Okugawa, Taketo Otsuka, Fujio Sasagawa, Masako Sakauchi, Masahisa Sato, Haruyoshi Ino, Masaya Ota, Miyako Kon, Akihiko Saitoh

**Affiliations:** Niigata University Graduate School of Medical and Dental Sciences, Niigata, Niigata, Japan; Niigata University Graduate School of Medical and Dental Sciences, Niigata, Niigata, Japan; Department of Pediatrics , Niigata University Graduate School of Medical and Dental Sciences, Niigata, Japan, Niigata, Niigata, Japan; Niigata University, Niigata, Niigata, Japan; Shin Pediatric Clinic, Niigata, Niigata, Japan; Ikarashi Pediatric Clinic, Niigata, Niigata, Japan; Okugawa Pediatric Clinic, Niigata, Niigata, Japan; Otsuka Pediatric Clinic, Niigata, Niigata, Japan; Sasagawa Pediatric Clinic, Niigata, Niigata, Japan; Sakauchi Pediatric Clinic, Niigata, Niigata, Japan; Sato Pediatric Clinic, Niigata, Niigata, Japan; Seikyou Pediatric Clinic, Niigata, Niigata, Japan; Ota Pediatric Clinic, Niigata, Niigata, Japan; Niigata Prefectural Institute of Public Health and Environmental Science, Niigata, Niigata, Japan; Niigata University, Niigata, Niigata, Japan

## Abstract

**Background:**

The coronavirus disease 2019 (COVID-19) messenger RNA (mRNA) vaccines were confirmed to be safe and effective, and the original BNT162b2 mRNA vaccine for children aged 6 months to 4 years was introduced in October 2022. However, COVID-19 vaccination coverage has been low in children, especially among those aged 6 months to 4 years. Few studies have evaluated vaccine effectiveness (VE) in this age group, and no data are available from Japan.

**Methods:**

A test-negative, case-control study was conducted between April and October in 2023 at 10 pediatric clinics in Niigata, Japan. We included patients aged 6 months to 4 years with upper respiratory tract symptoms who were tested for severe acute respiratory syndrome coronavirus 2 by rapid antigen testing or PCR assay. Cases and controls were defined as those with positive and negative test results, respectively. The recommended original BNT162b2 mRNA COVID-19 vaccine primary series comprised 3 doses, and a vaccine dose was recorded if at least 2 weeks had passed since the last vaccination. Odds ratios (OR) were calculated using a multivariable logistic regression model adjusted for sex, age, underlying disease, history of COVID-19, and outbreak period. VE was calculated as (1 − adjusted OR) × 100% and is presented with the 95% confidence interval (95% CI).

**Results:**

During the study period an outbreak of omicron subvariants XBB and EG.5 occurred between July and September. In total, 2253 patients were included—262 (11.6%) patients were the cases and the remaining 1991 patients were the controls; 181 (8.0%) patients received ≥3 vaccine doses, and 938 (41.6%) patients had a history of COVID-19. VE against COVID-19 was 62% (95% CI, 26–81%) after ≥3 doses. Three months after the last vaccine dose, VE against COVID-19 was 59% (95% CI, 13–81%) among patients who received ≥3 doses of COVID-19 mRNA vaccine.

**Conclusion:**

Among children aged 6 months to 4 years in Japan who received ≥3 doses of the original BNT162b2 mRNA COVID-19 vaccine, the vaccine was effective against COVID-19 infection by omicron subvariants for at least 3 months after the last dose. To ensure protection against COVID-19 in this vulnerable population, further follow-up study is necessary.

**Disclosures:**

**All Authors**: No reported disclosures

